# Supplementary material for: 9-N-n-alkyl Berberine Derivatives: Hypoglycemic Activity Evaluation
Source: Pharmaceutics. 2022 Dec 22;15(1):44. doi: 10.3390/pharmaceutics15010044 (PMC9865096; doi:10.3390/pharmaceutics15010044)
Supplement: Supplementary file 1 [file pharmaceutics-15-00044-s001.zip › pharmaceutics-2096042-supplementary.pdf]

Supplementary materials

9-N-n-alkyl berberine derivatives: hypoglycemic activity evaluation

Mikhail V. Khvostov[1*], Elizaveta D. Gladkova[1], Sergey A. Borisov[1], Marina S. Fedotova[1,3], Nataliya A. Zhukova[1], Mariya K. Marenina[1], Yuliya V. Meshkova[1], Nicolae Valutsa[2], Olga A. Luzina[1], Tatiana G. Tolstikova[1] and Nariman F. Salakhutdinov[1]

[1] N. N. Vorozhtsov Novosibirsk Institute of Organic Chemistry, Siberian Branch of the Russian Academy of Sciences, 9, Akademika Lavrentieva Ave., 630090 Novosibirsk, Russia;

[2] Department of Natural Sciences, Novosibirsk State University, Pirogova Str. 1, 630090 Novosibirsk, Russia

[3] V. Zelman Institute for Medicine and Psychology, Novosibirsk State University, Pirogova str. 1, 630090 Novosibirsk, Russia;

Spectral data

*9-(pentylamino)-2,3-methylenedioxy-10-methoxyprotoberberine chloride (**2a**)*

This compound was synthsized and described previously [10] Yield: 44%. ^{1}H NMR (400 MHz, DMSO-d$_6$): δ 10.10 (s, 1H, H-8), 8.68 (s, 1H, H-13), 7.88 (d, J = 8.6 Hz, 1H, H-9*), 7.75 (s, 1H, H-1), 7.46 (d, J = 8.6 Hz, 1H, H-10*), 7.06 (s, 1H, H-4), 6.42 (t, J = 5.8 Hz, 1H, NH), 6.15 (s, 2H, OCH$_2$O), 4.79 (t, J = 6.1 Hz, 2H, H-6), 3.95 (s, 3H, OCH$_3$), 3.54–3.60 (m, 2H, NH*CH$_2$*), 3.19 (t, J = 5.9 Hz, 2H, H-5), 1.57-1.65 (m, 2H, NHCH$_2$*CH$_2$*), 1.27-1.35 (m, 4H, NHCH$_2$CH$_2$*CH$_2$CH$_2$*), 0.87 (t, J = 6.9 Hz, 3H, CH$_2$*CH$_3$*). ^{13}C NMR (100 MHz, DMSO-d$_6$): δ 149.80, 147.93, 146.92, 137.56, 135.93, 133.34, (C-2, C-3, C-4a, C-10, C-12a, C-13a), 146.67 (C-8), 130.55, 120.91, 117.17 (C-8a, C-9, C-13b), 124.80 (C-13), 119.97 (C-12), 116.30 (C-11), 108.74 (C-4), 105.52 (C-1), 102.29 (OCH$_2$O), 57.28 (OCH$_3$), 55.13 (C-6), 47.60 (NHCH$_2$), 30.49, 28.90, 26.96, 22.25 (NHCH$_2$*CH$_2$CH$_2$CH$_2$*, C-5), 14.28 (CH$_2$*CH$_3$*). IR (KBr), ν/cm^{-1}: 3403.9 (NH). MS (ESI): m/z (M+) calcd for $C_{25}H_{29}N_2O_3$, 391.202 found: 391.202.

*9-(hexylamino)-2,3-methylenedioxy-10-methoxyprotoberberine chloride (**2b**, SHE 196)*

Yield: 52%. IR (KBr), ν/cm^{-1}: 3403.9 (NH). Other spectral data are the same as described previously [6]

*9-(heptylamino)-2,3-methylenedioxy-10-methoxyprotoberberine chloride (**2c**)*

Yield: 27%. ^{1}H NMR (400 MHz, DMSO-d$_6$): δ 10.10 (s, 1H, H-8), 8.68 (s, 1H, H-13), 7.88 (d, J = 8.7 Hz, 1H, H-9*), 7.75 (s, 1H, H-1), 7.46 (d, J = 8.7 Hz, 1H, H-10*), 7.06 (s, 1H, H-4), 6.40 (t, J = 6.07 Hz, 1H, NH), 6.15 (s, 2H, OCH$_2$O), 4.79 (t, J = 6.1 Hz, 2H, H-6), 3.95 (s, 3H, OCH$_3$), 3.54–3.60 (m, 2H, NH*CH$_2$*), 3.18 (t, J = 6.1 Hz, 2H, H-5), 1.57-1.64 (m, 2H, NHCH$_2$*CH$_2$*), 1.26-1.35 (m, 6H, NHCH$_2$CH$_2$*CH$_2$CH$_2$CH$_2$*), 0.85 (t, J = 6.9 Hz, 3H, CH$_2$*CH$_3$*). ^{13}C NMR (100 MHz, DMSO-d$_6$): δ 149.45, 147.58, 146.54, 137.24, 135.85, 133.00, (C-2, C-3, C-4a, C-10, C-12a, C-13a), 146.38 (C-8), 130.20, 120.57, 116.83 (C-8a, C-9, C-13b), 124.45 (C-13), 119.62 (C-12), 115.91 (C-11), 108.40 (C-4), 105.17 (C-1), 101.95 (OCH$_2$O), 56.92 (OCH$_3$), 54.74 (C-6), 47.24 (NHCH$_2$), 31.03, 30.43, 26.62, 26.00, 22.04 (NHCH$_2$*CH$_2$CH$_2$CH$_2$CH$_2$*, C-5), 13.88 (CH$_2$*CH$_3$*). IR (KBr), ν/cm^{-1}: 3357.6 (NH). MS (ESI): m/z (M+) calcd for $C_{25}H_{29}N_2O_3$, 405.217 found: 405.217.

*9-(decylamino)-2,3-methylenedioxy-10-methoxyprotoberberine chloride (**2d**)*

Yield: 40%. ^{1}H NMR (400 MHz, DMSO-d$_6$): δ 10.00 (s, 1H, H-8), 8.69 (s, 1H, H-13), 7.89 (d, J = 8.6 Hz, 1H, H-9*), 7.76 (s, 1H, H-1), 7.47 (d, J = 8.6 Hz, 1H, H-10*), 7.07 (s, 1H, H-4), 6.31 (t, J = 6.1 Hz, 1H, NH), 6.15 (s, 2H, OCH$_2$O), 4.78 (t, J = 6.2 Hz, 2H, H-6), 3.96 (s, 3H, OCH$_3$), 3.53–3.60 (m, 2H, NH*CH$_2$*), 3.15-3.20 (t, J = 6.2 Hz, 2H, H-5), 1.55-1.63 (m, 2H, NHCH$_2$*CH$_2$*), 1.18-1.34 (m, 14H, NHCH$_2$CH$_2$*CH$_2$CH$_2$CH$_2$CH$_2$CH$_2$CH$_2$CH$_2$*), 0.83 (t, J = 6.8 Hz, 3H, CH$_2$*CH$_3$*). ^{13}C NMR (100 MHz, DMSO-d$_6$): δ 149.76, 147.90, 146.92, 137.48, 135.89, 133.26 (C-2, C-3, C-4a, C-10, C-12a, C-13a), 146.68 (C-8), 130.52, 120.89, 117.10 (C-8a, C-9, C-13b), 124.59 (C-13), 119.98 (C-12), 116.30 (C-11), 108.73 (C-4), 105.49 (C-1), 102.28 (OCH$_2$O), 57.19 (OCH$_3$), 55.14 (C-6), 47.65 (NHCH$_2$), 31.61, 30.75, 29.31, 29.25, 29.11, 29.02, 26.91, 26.61, 22.41 (NHCH$_2$*CH$_2$CH$_2$CH$_2$CH$_2$CH$_2$CH$_2$CH$_2$CH$_2$*, C-5), 13.88 (CH$_2$*CH$_3$*). IR (KBr), ν/cм$^{-1}$: 3402.7 (NH). MS (ESI): m/z (M+) calcd for $C_{25}H_{29}N_2O_3$, 461.280 found: 461.280.

*9-(dodecylamino)-2,3-methylenedioxy-10-methoxyprotoberberine chloride (**2e**)*

Yield: 34%. ^{1}H NMR (400 MHz, DMSO-d$_6$): δ 10.00 (s, 1H, H-8), 8.70 (s, 1H, H-13), 7.89 (d, J = 8.6 Hz, 1H, H-9*), 7.76 (s, 1H, H-1), 7.47 (d, J = 8.6 Hz, 1H, H-10*), 7.07 (s, 1H, H-4), 6.32 (s, 1H, NH), 6.16 (s, 2H, OCH$_2$O), 4.79 (t, J = 5.9 Hz, 2H, H-6), 3.95 (s, 3H, OCH$_3$), 3.54–3.59 (m, 2H, NH*CH$_2$*), 3.17-3.21 (t, J = 5.9 Hz, 2H, H-5), 1.57-1.62 (m, 2H, NHCH$_2$*CH$_2$*), 1.18-1.36 (m, 18H, NHCH$_2$CH$_2$*CH$_2$CH$_2$CH$_2$CH$_2$CH$_2$CH$_2$CH$_2$CH$_2$CH$_2$*), 0.84 (t, J = 6.9 Hz, 3H, CH$_2$*CH$_3$*). ^{13}C NMR (100 MHz, DMSO-d$_6$): δ 149.79, 147.92, 146.83, 137.60, 135.90, 133.34, (C-2, C-3, C-4a, C-10, C-12a, C-13a), 146.74 (C-8), 130.52, 120.92, 116.17 (C-8a, C-9, C-13b), 124.81 (C-13), 119.95 (C-12), 116.17 (C-11), 108.74 (C-4), 105.51 (C-1), 102.30 (OCH$_2$O), 57.24 (OCH$_3$), 55.04 (C-6), 47.48 (NHCH$_2$), 31.62, 30.79, 29.38, 29.36, 29.32, 29.31, 29.13, 29.05, 26.96, 26.65, 22.42 (NHCH$_2$*CH$_2$CH$_2$CH$_2$CH$_2$CH$_2$CH$_2$CH$_2$CH$_2$CH$_2$CH$_2$*, C-5), 14.29 (CH$_2$*CH$_3$*). IR (KBr), ν/cм$^{-1}$: 3401.9 (NH). MS (ESI): m/z (M+) calcd for $C_{25}H_{29}N_2O_3$, 489.311 found: 489.311.

Histology

Histological examination demonstrated degenerative and necrotic changes in the AY mice' liver. Dystrophic changes: polymorphic lipid infiltration, focal necrosis of hepatocytes (infiltrated by macrophages and mononuclear leukocytes), hepatic bar dyscomplexation was detected in periportal areas in all animals (Figure 5). A lot of enlarged Kupffer cells were observed in the lumen of sinusoids. Glycogen was not detected. All these findings indicate the development of fatty hepatosis in AY mice. In the exocrine part of the pancreas no dystrophy or necrosis were detected. In the endocrine part pronounced hyperplasia of islet apparatus was detected (Figure 9). Brown adipose tissue analysis revealed a marked fat content increase in adipocytes. Large fat droplets merged with each other to form fat cysts (Figure 8). White adipose tissue examination discovered a prominent increase in the adipocytes' size and their fusion into fat cysts (Figure 7). Metformin treated AY mice showed improvement in the described metabolic abnormalities. The de-crease in the severity of dystrophic changes was found in the liver. Small vesicular lipid infiltration was primarily observed in the periportal areas. Inflammatory-necrotic, hemodynamic abnormalities were weakly pronounced (Figure 5). Periodic acid–Schiff staining (PAS staining) revealed glycogen in the form of dust-like granularity in hepatocytes of the central zones. In exocrine part of pancreas focal fatty dystrophy of acinocytes was detect-ed. In the endocrine part, the diameter of the islet apparatus was decreased (Figure 9). Metformin administration resulted in fat content decrease in brown and white adipose tissue: the reduction of fat's droplet size was observed (Figure 7,8). In AY mice treated with compound 2e, there was an increase in liver metabolic disorders. In these animals the development of total fatty hepatosis with signs of cholestasis, cytolysis of hepatocytes and distortion of architectonics in the form of liver beams discomplexation was observed. Polymorphic fatty dystrophy was registered in hepatocytes: fat vacuoles completely filled the cytoplasm, merged with each other, giving cells a reticular pattern, pushing nuclei to the periphery (Fig. 5, 6). These pathological changes were accompanied by massive cytolysis of hepatocytes and venous plethora. The sinusoidal lumen was sharply narrowed, and macrophage infiltration was observed periportally. Glycogen in the liver was not detected during Schick staining. No alterations (dystrophies, necrosis) were revealed in the exocrine part of pancreas. In the endocrine part, pronounced hyperplasia of the islet apparatus was observed similar to mice from the negative control group (Figure

9). Reduced fat content was observed in brown adipose tissue. Adipocytes were dominated by mainly small fat droplets (Figure 8). The size of adipocytes in the white adipose tissue also slightly decreased, indicating the activation of lipolysis processes (Figure 7).
